# Prognostic Value of *TLE1* Gene Expression in Patients with T-cell Acute Lymphoblastic Leukemia

**DOI:** 10.31557/APJCP.2021.22.5.1653

**Published:** 2021-05

**Authors:** Salah Aref, Mohamed Sabry El-Ghonemy, Doaa Mohamed Atia, Mohamed M Elbaiomy, Sherehan Adel Abdelsalam, Aya Tawfik, Ahmed EL-Sebaie

**Affiliations:** 1 *Hematology Unit, Clinical Pathology Department, Faculty of Medicine, Mansoura University, Mansoura, Egypt. *; 2 *Medical Oncology Unit, Mansoura University Oncology Center; Mansoura; Egypt. *; 3 *Public health at Community Medicine Department, Faculty of Medicine, Mansoura University, Egypt.*; 4 *Clinical pharmacy, Faculty of pharmacy, Mansoura University, Egypt. *

**Keywords:** TLE1, T-ALL, Prognosis, DFS- OS

## Abstract

**Background:**

Transducin-like enhancer of split 1 (*TLE1*( is a member of the TLE family of transcriptional co-repressors that control the transcription of a wide range of genes. The aim of this study was to evaluate the prognostic role of *TLE1* gene expression in patients with T-cell acute lymphoblastic leukemia (T-ALL).

**Method:**

This study was conducted on 97 newly diagnosed T-ALL patients admitted to the Mansoura University oncology center (59 males and 38 females) with median age (33 years) in addition to 102 apparently healthy individuals served as a control group. *TLE1* gene expression was measured in both patients and control groups by real time – PCR. The calculation of relative gene expression was done using the ΔΔCt method.

**Results:**

TEL1 gene expression was significantly down regulated in T-ALL cases (median 2.83) as compared to controls (median 84.65) (p < 0.001). The low TEL1 gene expression was significantly associated with CNS infiltration, non-remission and higher relapse rate (p< 0.001, 0.001 and 0.023 respectively). Likewise, Low TEL1 gene expression was significantly associated with shorter OS and DFS (P= 0.012 and 0.011 respectively). Furthermore, Low TEL1 gene expression was considered as risk predictor of relapse with OR 3.636(CI.1.422-9.295) (P =0.007); and OR 0.803(CI. 0.609-0.96) (P=0.021) and independent predictor of T-ALL patient’s outcome with OR 0.619 (CI. 0.44-0.872) (P=0.006).

**Conclusion:**

*TLE1* gene expression was significantly down regulated in T-ALL cases as compared with controls. Low *TLE1* expression is independent predictor of the T-ALL patient’s outcome.

## Introduction

Acute lymphoblastic leukemia (ALL) is a malignant transformation and proliferation of lymphoid progenitor cells in the bone marrow, blood and extra-medullary sites. ALL is the most common childhood cancer. The ALL is characterized by chromosomal anomalies and genetic alterations implicated in differentiation and proliferation of lymphoid precursors. In adults, 75% of cases develop from B-cell precursors while 25% from T-cell precursors (Terwilliger and Abdul-Hay, 2017).

The pathogenesis of ALL involves the abnormal proliferation and differentiation of a clonal population of lymphoid cells. The maturation process of lymphocytes from pluripotent hematopoietic stem cells in the bone marrow is mainly controlled by the activation of transcription factors and selection through functional signal transduction. ALL is considered a group of B/T-precursor lymphoid cell malignancies that blocks differentiation and drives aberrant cell proliferation and survival (Zhou et al., 2012). In the majority of cases, it appears as a de novo malignancy in previously healthy individuals. Chromosomal aberrations are characteristic in ALL, but are not sufficient to generate leukemia such as t(12; 21) (ETV6-RUNX1), t (1;19) (TCF3-PBX1), t (9;22) [BCR-ABL1 (Philadelphia)] and rearrangement of MLL (Mullighan et al., 2009). 

Transducin-like enhancer of split 1 (*TLE1*) is a member of the Groucho (Gro)/TLE family of transcriptional co-repressors located at chromosome 9q21.32. *TLE1* regulates the transcriptional activity of a wide range of genes and has been implicated in embryogenesis, hematopoiesis, and neuronal and terminal epithelial differentiation (Agarwal et al., 2015). As a co-repressor, the TLE1 protein does not bind to DNA directly but is recruited to target gene(s) by direct interaction with DNA-binding transcription factors to form large multi-protein complexes. Alternatively, the TLE1 may interact with chromatin through its interactions with the amino-terminal tail of histone H3 (Yao et al., 2014). TLE1 protein consists of five conserved domains: Q (glutamine-rich domain), GP (glycine/proline-rich domain), CcN (phosphorylation sites for Cdc2 and kinase2), SP (Serine/proline rich domain), and WD40 (WD40-repeat domains) (Dastidar et al., 2012). The CcN and SP domains are less conserved and regulate the sub-cellular localization, phosphorylation state and transcriptional repression activity of *TLE1*. The Q and WD40 domains are required for *TLE1* function. In particular, the WD40-repeat containing domain is involved in; signal transduction, transcription regulation, cell cycle control, autophagy and apoptosis (Stirnimann et al., 2010). This domain interacts with a large number of transcriptional regulator genes involved in several signal transduction pathways for cell fate specification, proliferation and migration, including the Notch, Wingless/Wnt and Dpp/BMP/TGF-β signaling pathways (Turki-Judeh and Courey, 2012). Recently, the critical role of *TLE1* has been established in the progression of various tumors as; synovial sarcoma, gastric carcinoma, hematological malignancies and breast cancer (Liu et al., 2019). The NF-kB Notch and Wnt/b-catenin signaling pathways have been revealed to be involved in *TLE1*- modulation of tumors (Ramasamy et al., 2016). Up till now; there are no genetic biomarkers that can predict T-ALL patient’s outcome or treatment response.

The aim of this study was to address the prognostic value of *TLE1* gene expression in patients with T cell acute lymphoblastic leukemia.

## Materials and Methods

After approval of the Local Ethics Committee of Mansoura University, Faculty of Medicine and obtaining written informed consent from all patients, this study was conducted on 97 newly diagnosed patients of T-ALL admitted to the Mansoura University Oncology center (MUOC). In addition, 102 healthy individuals were served as a control group.

All laboratory procedures were performed in the clinical pathology laboratories of MUOC at the time of T-ALL diagnosis before induction therapy.

Patients received treatment with Hyper-CVAD regimen (Kantarjian et al., 2000) or Berlin-Frankfurt-Münster (aBFM) regimen (Rytting et al., 2014). In patients with Philadelphia chromosome positive ALL, Tyrosine kinase inhibitors (TKIs) (Imatinib, Nilotinib or Dasatinib) were offered. After induction of remission, maintenance treatment with vincristine, corticosteroid, weekly methotrexate and 6-mercaptopurine (6-MP) daily for 2–3 years was added. TKIs to maintenance regimen (in Ph positive patients).

All patients were subjected to through history taking, physical examination searching for purpuric eruption, ecchymosis, lymphadenopathy, and organomegaly. Routine investigations were performed to patients and controls including routine complete blood count (CBC), liver function tests, renal function test, and serum lactate dehydrogenase. Workup for T-ALL diagnosis: Based on morphological evaluation for the peripheral blood and bone marrow smears (Blast cells ≥20%). Immunophenotyping was performed using a BD flow-cytometer (Counter Epics XL flow cytometer PN 2372238B counter corporation, Miami, Florida 33196, USA) device to confirm diagnosis.

*TLE1 gene expression analysis*

Two milliliters of blood were collected in a tube containing an EDTA for genomic RNA extraction. cDNA was obtained from 1.5 µg of total RNA using Super Script II (Thermo Fisher) and ampliﬁed by real time-PCR using the following primers: The *TLE1* (F); 5′-CCTCCTACACAGCAGCAGTT-3′ and (R); 5′-TCTGCATCGTGGTGCTTCTT-3′. The GAPDH was used as the reference gene, and the primers were: (F); 5′-CGGAGTCAACGGATTTGGTCGTAT-3′ and (R); 5′-AGCCTTCTCCATGGTGGTGAAGAC-3′. This protocol pass through following steps; pre-incubation: 95°C, 10 min; ampliﬁcation: 40 cycles of 95°C, 10 s; 60°C, 30 s; 72°C, 10 s. The calculation of relative gene expression was done using the ΔΔCt method.

*Statistical analysis*

*Sample Size*

The power of the study was calculated by the G*Power software (Version 3.1.9.2). At sample size of 97 and 102 for cases and control groups respectively, achieved power was 86.1% at the alpha probability of 0.05, using medium effect size of 0.44. For this purpose, T-test model means: Wilcoxon-Mann Whitney test (two groups) were performed. The comparative Ct method was used for gene expression quantification. Gene expression levels for each sample were normalized to the expression level of the housekeeping gene encoding the housekeeping enzyme within a given sample (ΔCt). Results were evaluated by using 2^-^^ΔΔ^^CT^ method as relative gene expression values.

*Endpoints definitions*

Overall survival is defined as the time from diagnosis until death or end of the study. DFS is defined as the time from remission until disease relapse, death or end of the study. Cumulative incidence of relapse: is a measure of the occurrence of ALL relapse over a stated period of time.

The collected data were analysed by Statistical package for Social Science (IBM Corp. Released, 2017. IBM SPSS Statistics for Windows, Version 25.0. Armonk, NY: IBM Corp.). Mann Whitney Test was used to detect the statistically significant differences between two study groups. Chi-Square test was performed to examine the relationship between two qualitative variables. Logistic regression analysis was used for prediction of risk factors, using generalized linear models. Kaplan–Meier test was used for survival analysis and the statistical significance of differences among curves was determined by Log-Rank test. Cox regression analysis of factors potentially related to survival was performed to identify which independent factors might jointly have a significant influence on survival. A p value is considered significant if < 0.05.

## Results

The present study was conducted on 97 adult T-ALL. Their median age was 33 years, ranged from (19 to 78 years). They were 59 (60.8 %) males and 38 (39.2 %) females. In addition, 102 healthy individuals as control group were subjected of matched age and gender.

**Figure 1.**
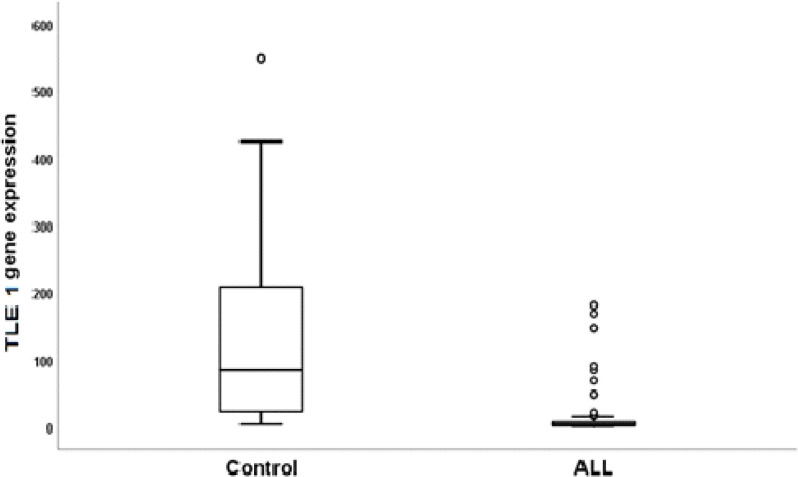
*TLE1* Expression in Studied Patients with T-ALL vs Controls

On comparison of *TLE1* expression between patients with T-ALL and controls, we were found that TEL1 gene expression was significantly down regulated in T-ALL cases (median *TLE1* expression: 2.83) as compared to controls (median *TLE1* expression : 84.65) (P < 0.001) (Figure 1).

The T-ALL patients were categorized into 2 subgroups; T-ALL with high expression (>median; n=48) and T-ALL with low expression (≤median; n=49). Comparison of the studied parameters in T-ALL with low *TLE1* expression and T-ALL subgroup with high expression; the analysis showed that the low TEL1 gene expression was significantly associated with higher frequency CNS infiltration, non-remission and higher relapse rate (P < 0.001, < 0.001 and 0.023 respectively). No significant differences were found between low and high *TEL1 *expression regarding age, gender, laboratory data (P= 0.653) (Table 1).

Cox regression analysis was performed for prediction of OS using LDH, high cytogenetic risk and *TEL1* gene expression as covariates. High LDH, CNS infiltration, low *TEL1* gene expression was associated with significantly shorter OS in univariable analysis (P=0.029, 0.036 and 0.012 respectively). However, in multivariable analysis, only down regulated *TEL1* gene expression was considered as poor prognostic factor for shorter OS (P=0.006) (Table 2).

When Cox regression analysis was conducted for prediction of DFS, it showed that CNS infiltration and low *TEL1* gene expression were considered as poor prognostic factors for shorter DFS in both univariable (P= 0.024, 0.034 and 0.011 respectively) and multivariable (P=0.002, 0.035 and 0.002 respectively) analyses (Table 2).

Logistic regression analysis was conducted to predict the relapse occurrence using the same parameters. Low *TEL1* gene expression was considered as risk predictors of relapse in univariable (P=0.001 and 0.032 respectively) and multivariable (P=0.007 and 0.021 respectively) (Table 2).

**Figure 2:**
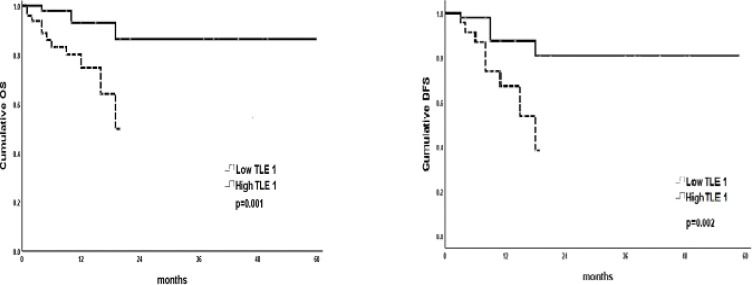
Kaplan-Meier Curve to Test the Impact of *TLE1* Gene Expression (High vs Low) on OS and DFS in Patients with T-ALL. T-ALL patients have high TLE1 expression showed longer OS and DFS as compared to those have low *TLE1* expression (P=0.001 and 0.002 respectively)

Figure 2 showed the T-ALL patients with low *TLE1* expression have short OS and shorter DFS as compared to subgroup of T-ALL patients with high TEL1 group (P0.001; 0.002 respectively).

**Figure 3:**
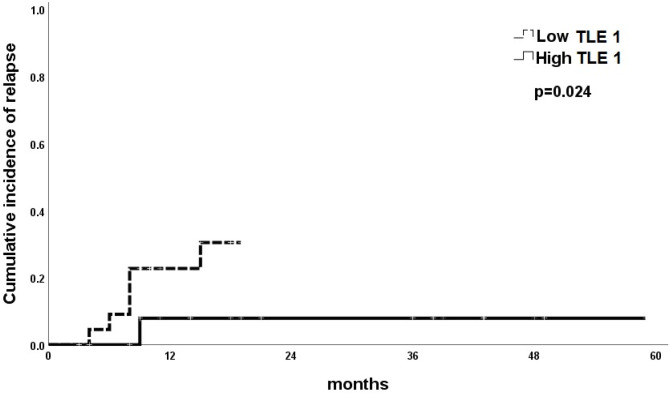
Comparison of the Cumulative Incidence of Relapse between Patients with T-ALL Subgroup who have Low versus who have High *TLE1* Gene Expression.

Comparison the cumulative incidence of relapse (CIR) between the T-ALL patients with low and high TEL1 gene expressions revealed that the CIR was significantly higher subgroup of T-ALL patients with low TEL1 expression as compared to those with high TEL1 expression (P=0.024) (Figure 3).

**Table 1:** Comparison of Studied Parameters between Low and Hhigh *TLE1* Levels at Diagnosis in T-ALL Cases

		Low *TEL1*		High *TEL1*		p
		N=(49)		N=(48)		
Age (years)	Median, IQR	36	27-53	32.5	19.25-49	0.102
Males	N, %	27	55.10%	32	66.70%	0.243
Females	N, %	22	44.90%	16	33.30%	
TLC (X10^9^/L)	Median, IQR	23	4.1-71.25	45	6.9-81	0.139
Hb (g/dL)	Median, IQR	8.4	7.7-9.75	8.7	7.3-9	0.298
RBCS	Median, IQR	3.3	2.98-3.8	2.99	2.6-3.3	0.105
Platelets (X10^9^/L)	Median, IQR	25	17.5-97.8	57.5	18-121.95	0.321
BM blasts (%)	Median, IQR	80	61-90	85	80-90	0.125
LDH (U/L)	Median, IQR	789	423-2171	607	432-987	0.367
CNS infiltration	N, %	27	51%	3	6.20%	0.001
Remission	N, %	23	46.90%	46	95.80%	<0.001
Non remission	N, %	26	53.10%	2	4.20%	
Relapse	N, %	6	12.20%	3	6.30%	0.023

IQR, Interquartile range

**Table 2:** Regression Analysis for Prediction of OS, DFS and Relapse in Patients with T-ALL

	Univariable		Multivariable	
	P value	HR (95% CI)	P value	HR (95% CI)
Cox regression analysis for prediction of OS				
LDH	0.029	1.034 (1.003-1.076)	0.936	1.043 (0.957-1.342)
CNS infiltration	0.036	2.986 (1.077-8.279)	0.098	0.357 (0.10-1.209)
TLE1	0.012	0.702 (0.533-0.925)	0.006	0.619 (0.44-0.872)
Cox regression analysis for prediction of DFS.				
LDH	0.413	1.023 (0.947-1.079)		
CNS infiltrations	0.024	3.218 (1.164-8.901)	0.002	1.028 (1.003-1.262)
TLE1	0.011	0.746 (0.595-0.936)	0.002	0.373 (0.201-0.693)
Logistic regression analysis for prediction of Relapse.				
LDH	0.192	0.993 (0.988-1.012)		
CNS infiltration	0.265	1.551 (0.717-3.354)		
TLE1	0.032	0.774 (0.591- 0.913)	0.021	0.803 (0.609-0.96)

HR, hazard ratio; OR, odds ratio; CI, confidence interval.

## Discussion

In the current study; we found that *TLE1* gene expression was significantly down regulated in T-ALL cases (median 2.83) as compared to controls (median 84.65). This finding is similar with the results reported by Brassesco et al 2018 who found that *TLE1* expression levels were significantly down regulated in pediatric T-ALL patients (n=60) in relation to normal controls.

On comparison of studied parameters between low and high *TLE1* levels in T-ALL cases, it showed that the low *TLE1* gene expression was significantly associated with higher frequency of high CNS infiltration, non-remission and higher relapse rate. Similar findings were reported in pediatric T-ALL (Brassesco et al., 2018). TLE proteins can down-regulate the expression of transcriptional activators as well as enhance the effects of transcriptional repressors. In addition, they can convert transcriptional activators into repressors. It acts mainly as an effector on crucial signaling pathways such as Wnt, Notch and NF-κB determining cell fate. Thus, they have critical functions in growth and development. Recently, many studies have focused on the roles of TLE proteins in apoptosis and malignant progression (Wang et al., 2004; Yuan et al, 2017). Moreover; Fraga et al., (2008), documented that in hematological malignancies such as diffuse large B-cell lymphoma and AML, reintroduction of *TLE1* into leukemia or lymphoma cells resulted in growth inhibition in vitro and in vivo. In contrast, depletion of *TLE1* in leukemic cells enhanced tumor growth, indicating epigenetic inactivation of *TLE1* promoted the development of hematological malignancies by disrupting cell differentiation and growth-suppressive pathways.

The *TLE1* gene functions as a repressor of AML, which regulates the hematopoietic cell differentiation and proliferation through binding to the Runt domain and the C-terminus of AML1 including the VWRPY motif (Imai et al, 1998).

In the present study, the Cox regression analysis was conducted for prediction of OS, DFS and T-ALL relapse using LDH, high CNS infiltration and *TEL1* gene expression as covariates. The statistical analysis results indicate that high LDH, high CNS infiltration, low *TEL1 *gene expression was associated with significantly shorter OS in univariable analysis. However, in multivariable analysis, only down regulated *TEL1* gene expression was considered as poor prognostic factor for shorter OS. For prediction of DFS, the results indicated that high CNS infiltration and low *TEL1* gene expression were associated shorter DFS in both univariable and multivariable analyses. Also, for prediction of relapse we found that low *TEL1* gene expression was considered as independent predictor of relapse. Similar results were reported by Abou Dalle et al., (2019).

The prognostic value of *TLE1* expression was evaluated in other types of cancer. Wang et al., (2020) showed that high *TLE1* expression was significantly associated with better disease-specific survival (DSS), in patients with Pancreatic Ductal Adenocarcinoma. Also, Lee et al., (2016) showed that *TLE1* expression is a good indicator of prognosis in patients with gastric cancer both OS and DFS. *TLE1* deficiency also resulted in lung hypoplasia, decreased overall survival, and enhanced transplanted tumor growth (Ramasamy et al., 2016). On other hand, the study of lee et al 2017 in patients with breast cancer demonstrated insignificant correlation be¬tween *TLE1* expression and disease-free survival (DFS) (p=0.167) or overall survival (OS).

In conclusion, low *TLE1* expression in T-ALL patients is associated with poor T-ALL patients’ outcome. So; *TLE1* gene expression could be a valuable biomarker for risk stratification of adult T-ALL patients.

## Author Contribution Statement

Conceptualization: Salah Aref; Data interpretation: Mohamed Sabry El-Ghonemy, Doaa Mohamed Atia; Formal analysis: Sherehan Adel Abdelsalam, Aya Tawfik; Investigation: Doaa Mohamed Atia, Elbaiomy MA; Clinical assessment: Elbaiomy MA; Methodology: Ahmed EL-Sebaie, Mohamed Sabry El-Ghonemy; Resources: All authors; Manuscript writing: All authors. 
